# Development of a brief form of the Chinese version of the Odor Awareness Scale(OAS-B): A confirmatory factor analysis approach to item reduction

**DOI:** 10.1371/journal.pone.0334168

**Published:** 2025-10-09

**Authors:** Binfeng Zhang, Xiuxia Li, Mei He, Shanggang Huang, Shuling Huang, Lu Wang, Lei Cao, Hao Xu, Guanghui Nie

**Affiliations:** 1 Nanning Fifth People’s Hospital, Nanning, China; 2 Department of Psychology, Guangxi Medical University, Nanning, China; University of Kelaniya, SRI LANKA

## Abstract

**Objective:**

The Odor Awareness Scale is a questionnaire to assess individual differences in awareness of odors in the environment, the Chinese version of which has a long length with 27 items, and its psychometric properties do not meet an excellent level. Hence, this study aimed to develop a brief form of the Chinese version of the Odor Awareness Scale(OAS-B) with sufficient psychometric properties.

**Materials and methods:**

A total of 978 participants from a college were recruited, who were randomly allocated into two groups for item reduction(N = 492) and scale validation(N = 486). Confirmatory factor analysis was utilized to remove items identified as information overlaps. After removing overlapping items, the brief scale was validated in the other subgroup for reliability and validity. The criterion-related validity was measured by correlation analysis between OAS-B and the Social Odor Awareness Scale(SOS), the Body Odor Sniffing Questionnaire(BOSQ), and the Generalized Anxiety Disorder Scale (GAD-7).

**Results:**

Removing 12 overlapping items, OAS-B holds 15 items with three subscales. The model fit indices for the OAS-B were: $\chi^{\text{2}}/df$ = 2.594, RMSEA = 0.057, CFI = 0.938, TLI = 0.925, SRMR = 0.046. The Cronbach’s α coefficient for OAS-B was 0. 868. The Pearson correlations between OAS-B and SOS, BOSQ, and GAD-7 were 0.518 (*p* < 0.01), 0.410 (*p* < 0.01) and 0.165 (*p* < 0.01).

**Conclusion:**

OAS-B has a short length with sufficient psychometric properties, which could be applied to measure odor awareness in general population.

## 1. Introduction

The sense of smell is woven into the fabric of everyday life. From dawn to dusk, airborne molecules continuously impinge on the nasal epithelium, subtly steering decisions, biasing attention, and coloring mood long before their presence is verbally acknowledged [1–5]. Odor awareness denotes the idiosyncratic weight that people assign to these chemosensory events when they form attitudes or choose actions [6]. Operationally, it captures how readily an individual detects, encodes, and comments on olfactory information without external prompting [7–9]. High-awareness observers inhabit an olfactorily salient world in which scents effortlessly “pop out,” whereas low-awareness individuals register the same vapors only when attention is explicitly redirected toward them. Behaviourally, this trait gradient predicts large differences in olfactory acuity: low-scorers exhibit markedly elevated thresholds, diminished identification accuracy, and poorer discrimination relative to their high-scoring counterparts [6,10]. They also recall fewer previously encountered odors and require higher concentrations for recognition [11]. Consequently, odor awareness can be leveraged as a rapid proxy for global olfactory competence [9].

Accumulating evidence further indicates that reduced chemosensory function often accompanies psychiatric conditions, implicating olfactory variables in the broader landscape of mental-health vulnerability [12–16]. In some case-control studies, it has been found that individuals with panic disorder have a higher odor awareness than healthy individuals in the control group [17,18]. Other cross-sectional studies have also reported that odor awareness has a positive relationship with anxiety symptoms and a negative relationship with alexithymia in general population [19,20]. These evidences above demonstrate the importance and research value of odor awarenness.

The Odor Awareness Scale(OAS) is a tool used to measure odor awareness, developed by Smeets et al. [6] among university students, with a length of 32 items, and there is a Chinese version revised by Zhang et al. [20], comprising 27 items. These two versions are valid but too long to implement for routine administration, as they place a heavy burden on respondents, necessitating the development of a shorter and more concise scale to suffice various research and applications [21–23]. Meanwhile, the Chinese version of OAS, revised from the original OAS, achieved a good model fit in confirmatory factor analysis(CFA) after setting 10-item error correlations, which is a data-driven technique. Although setting error correlations is common in practice [24], it has drawbacks of leading to non-independent errors which suggest that after extracting the common factor, the items are still influenced by unexplained factors other than the measured latent variable, resulting in information overlap among items. This redundancy diminishes the psychometric properties of the scale to some extent. Therefore, many statisticians advocate caution in setting error correlations and do not recommend it as a priority choice [24–27]. One solution to this issue is deleting one of the correlated error items, which also aligns well with the strategy of reducing scale items.

Both the original OAS and the Chinese version utilize factor analysis techniques to establish a multilevel conceptual model. Like many psychological concepts, odor awareness comprises three levels: the basic level being odor awareness, and the second level being dimensions, with the Chinese version containing three dimensions or factors(i.e., odor sensitivity, odor impact, and odor attention). The third level is the indicator level, which refers to the scale items. The OAS assessment method involves summing the scores, with a higher total score indicating a greater level of odor awareness, suggesting the functional equivalence of the indicator level, meaning that several items reflect the same underlying construct, which implies the substitutability of items [28]. Hence, the equivalence of scale items creates the feasibility of item reduction.

Collectively, the objective of this study is to develop a brief form of the Chinese version of the Odor Awareness Scale (OAS-B) among college students. This revised version seeks to facilitate practical application while enhancing the psychometric properties of the scale, thereby improving its overall quality.

## 2. Materials and methods

### 2.1. Participants

From November 10, 2023, to December 31, 2023, participants were recruited by distributing anonymous questionnaires at a medical university in Guangxi, primarily during class breaks and also in other locations around the campus, such as sidewalks, cafeterias, and plazas. After explaining the content and purpose of the questionnaire, students voluntarily participated in the study. Inclusion criteria required participants to be aged between 18 and 45 to minimize age-related olfactory variability [29]. Exclusion criteria included: (1) anosmia; (2) acute respiratory infections; (3) chronic obstructive pulmonary disease or nasal surgery; (4) other neurological diseases affecting olfactory function, such as Parkinson’s disease and epilepsy. The final sample comprised 978 participants, with 270 males (27.6%) and 708 females (72.4%), aged 21.898 ± 2.056 years, ranging from 18 to 35 years. The sample was randomly divided into two subsamples: Sample A and Sample B. Sample A, consisting of 492 participants (116 males, 23.6%; 386 females, 76.4%; aged 21.953 ± 2.081 years, ranging from 18 to 35 years), was used for the item reduction procedure. Sample B, consisting of 486 participants (154 males, 31.7%; 332 females, 68.3%; aged 21.842 ± 1.998 years, ranging from 18 to 32 years), was used to validate the reliability and validity of the shortened scale. In terms of sample size, MacCallum [30] recommended sufficient sample sizes in factor analysis as follows: 100 = poor, 200 = average, 300 = good, 500 = very good, and >1000 = perfect. Thus, the subsample sizes of this study nearly meet the level of “very good”, indicating a sufficient sample size.

The implementation of this study complied with the principles of the Helsinki Declaration and was approved by the Medical Ethics Committee of Guangxi Medical University (protocol no. KY0243). All participants provided written informed consent to participate in the study.

### 2.2. Measures

#### 2.2.1. The Chinese version of the Odor Awareness Scale (OAS).

OAS [20] is designed to assess individual differences in the awareness of environmental odors, reflecting how individuals process olfactory information, respond to stimuli, and their level of attention to odors. Examples of items include: “ When you walk through the woods, do you pay attention to the odors surrounding you?” and “ Do you notice food odors emanating from houses when you are outdoors?”. The scale consists of three subscales with 27 items: odor sensitivity, odor impact, and odor attention. Odor sensitivity, which includes 8 items, refers to the ability to detect or differentiate various smells. Odor impact, comprising 13 items, addresses the influence of odors on emotions, behavior, and cognition. Odor attention, with 6 items, measures the degree of attention to environmental smells. Each item is rated on a 5-point Likert scale, with higher total scores indicating greater odor awareness. In this study, the Cronbach’s α coefficient for the OAS was 0.909.

#### 2.2.2. The Social Odor Awareness Scale (SOS).

SOS, developed by Dal Bo et al. [31], measures awareness of body odors, with items such as “When I smell the odor of someone I care about, I feel relaxed.” Social odor refers to body odor. The scale consists of three subscales with a total of 12 items. The three subscales are familiar social odor, romantic partner social odor, and stranger social odor, each containing 4 items. Each item is rated on a 5-point Likert scale, with higher total scores indicating greater social odor awareness. SOS was utilized as a measure of convergent validity for its odor-related construct.

For this study, the English version of the SOS was translated into Chinese: the original scale was translated into Chinese by a bilingual psychology professor and a graduate student, and then distributed to 10 graduate students for preliminary testing. Based on their feedback, the scale was revised and back-translated into English by an English teacher familiar with psychology to ensure fidelity to the original text. This resulted in the Chinese version of the SOS used in this study. CFA showed good fit indices for the Chinese SOS: $\;\chi^{\text{2}}/df\;$= 6.687, RMSRA = 0.076, CFI = 0.931, TLI = 0.905, SRMR = 0.071. The Cronbach’s α coefficient for the scale was 0.802, indicating good reliability and validity of the SOS in this study.

#### 2.2.3. The Body Odor Sniffing Questionnaire(BOSQ).

The BOSQ assesses individuals’ behaviors related to sniffing body odors [32], with items such as “Have you ever intentionally smelled a family member’s scent?” The BOSQ consists of 17 items assigned into three subscales: others’ body odor, self-common body odor, and self-private body odor. Each item is rated on a 4-point Likert scale, with higher total scores indicating a higher frequency of sniffing one’s own or others’ body odors. In this study, the Cronbach’s α coefficient for BOSQ was 0.892. BOSQ was utilized as a measure of convergent validity for its odor-related construct.

#### 2.2.4. The Generalized Anxiety Disorder Scale (GAD-7).

GAD-7 is a screening tool used to assess the severity of generalized anxiety symptoms [33]. The GAD-7 comprises 7 items designed to evaluate how frequently respondents have been bothered by 7 different problems over the past two weeks, with each item rated on a 4-point scale. Higher total scores indicate greater levels of anxiety. In this study, we used the Chinese version of the GAD-7 translated and revised by Xiaoyan et al. [34]. The Cronbach’s α coefficient for the GAD-7 in this study was 0.900. GAD-7 was utilized as a measure of discrimination validity because of the different construct from OAS.

### 2.3. The sequential item reduction strategy

Although there is no standardized method for shortening the length of scales, various studies have proposed a set of principles or recommendations to improve the quality of scale reduction [21–23]. One rigorous approach to item reduction is the sequential item reduction strategy proposed by Goetz et al. [21]. This strategy emphasizes maintaining the underlying conceptual model of the scale without diminishing the psychometric properties in terms of reliability and validity while sequentially reducing the items. Goetz et al. recommend CFA for item reduction, which in this study included three steps:

#### 2.3.1. Step 1: Testing the existing model.

Conduct CFA on the original OAS to establish a baseline model for comparison with the modified versions. This step also involves reporting the internal consistency reliability of the scale.

#### 2.3.2. Step 2: Identifying overlapping items for removal.

Remove items with information overlap. Items are identified based on modification indices (MI > 10), expected parameter changes (EPC > 0.1), and standardized residual covariances (SRC > 0.2) [35–37]. MI highlights fixed parameters whose release would markedly improve model fit, signaling possible cross-loadings or residual covariances; EPC quantifies the anticipated size of those freed parameters, helping judge practical significance; and SRC reveals item pairs whose observed relationship is poorly reproduced by the model, flagging content overlap [38]. Once items with overlap are identified, decisions on which items to remove are made considering their content and theoretical relevance to avoid misrepresenting the scale’s latent constructs due to item reduction. Items with standardized factor loadings less than 0.4 should also be removed [39]. Each factor should retain at least three items [35].

#### 2.3.3. Step 3: Validating the shortened scale in a separate sample.

Validate the shortened version of the OAS (OAS-B) in a different sample, reporting its reliability and validity, including internal consistency, construct validity, convergent validity, and discriminant validity. Construct validity refers to the extent to which the test measures the theoretical construct it is intended to measure and is assessed by CFA. Convergent validity refers to the relationships with other tests measuring similar constructs, evaluated by correlating the OAS-B with SOS, and BOSQ. Discriminant validity refers to the relationships with tests measuring different constructs, assessed by correlating the OAS-B with the GAD-7.

### 2.4. Statistics

Statistical analyses were conducted using SPSS 27.0 and Mplus 8.3 software. In Sample A, internal consistency reliability and construct validity of OAS were assessed. CFA utilized multiple fit indices to evaluate model fit: Normed Chi-square ($\chi^{\text{2}}/df$) < 3, Comparative Fit Index (CFI) ≥ 0.9, Tucker-Lewis Index (TLI) ≥ 0.9, Standardized Root Mean Squared Residual (SRMS) < 0.8, and Root Mean Square Error of Approximation (RMSEA) < 0.8. Achieving these standards indicates good model fit [40–43]. Following item reduction in Sample A to obtain the OAS-B, the reliability and validity of OAS-B were then validated in Sample B. Internal consistency was evaluated using Cronbach’s α coefficient, while convergent and discriminant validity were assessed through Pearson correlation analysis. The significance level was set at α = 0.05.

## 3. Results

### 3.1. Item reduction

#### 3.1.1. Step 1: Testing the existing model.

**Fig 1:**
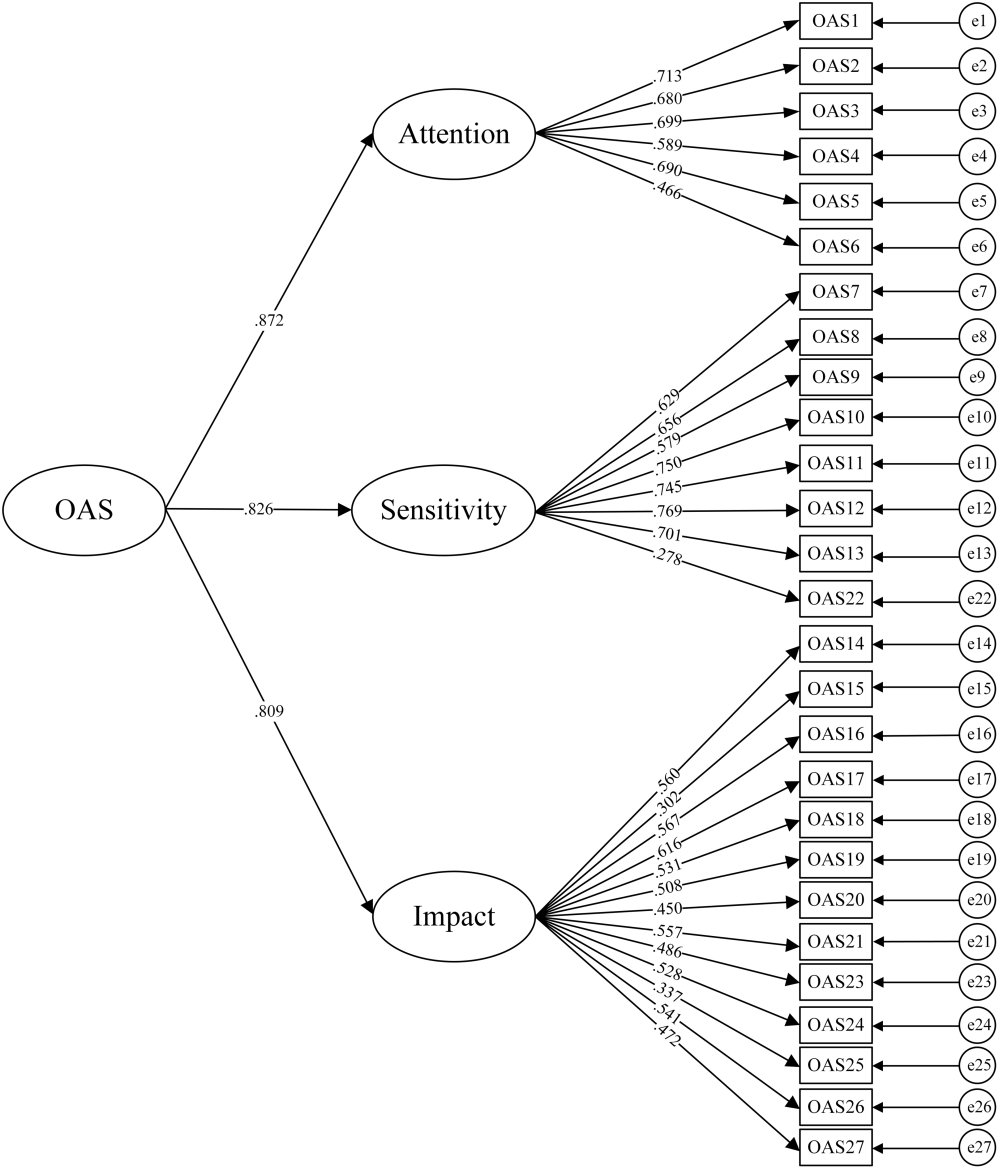
Confirmatory factor structure of the Chinese version of the Odor Awareness Scale.

The item reduction procedure was conducted using Sample A. The results showed that the Cronbach’s α coefficient for the OAS was 0.907, with the Cronbach’s α coefficients for the three subscales being: odor attention (0.796), odor sensitivity (0.867), and odor impact (0.827), indicating good internal consistency for OAS. The factor model of OAS is a second-order model, so this study adhered to the original model by applying the second-order model for the item reduction procedure. CFA revealed the following fit indices for the OAS: $\chi^{\text{2}}/df\;$= 3.629, RMSEA = 0.073, CFI = 0.811, TLI = 0.794, SRMR = 0.068. The standardized factor loadings for items 15, 22, and 25 were below 0.4, with values of 0.302, 0.278, and 0.337, respectively (see Fig 1). The results of the CFA indicated that the structural validity of OAS was moderate and did not reach an excellent level.

#### 3.1.2. Step 2: Identifying overlapping items for removal.

After identifying items with information overlap based on the indicators, decisions on item removal were made according to the fit of items to the conceptual model and dimensions, the factor loadings, and the number of overlapping items. Ultimately, 12 items were removed including 3 items with standardized factor loadings below 0.4, resulting in the 15-item OAS-B which holds three subscales——odor attention subscale containing 5 items, odor sensitivity subscale containing 4 items, and odor impact subscale containing 6 items. The model fit indices for the OAS-B were: $\chi^{\text{2}}/df\;$= 1.849, RMSEA = 0.042, CFI = 0.965, TLI = 0.958, SRMR = 0.039, indicating that OAS-B has good structural validity. The Cronbach’s α coefficient for OAS-B was 0.855, with Cronbach’s α coefficients for the subscales being: odor attention (0.754), odor sensitivity (0.816), and odor impact (0.723), demonstrating good internal consistency for OAS-B. Details of the removed items and the criteria for exclusion are provided in Table 1.

**Table 1:** OAS-B: item content and reasons for item exclusion.

Items	Reasons for removal
When you walk through the woods, do you pay attention to the odors surrounding you?	Retained
When someone is busy in the kitchen, do you notice the odor of the food being prepared?	Removed due to overlap with item 3 (MI = 15.300, EPC = 0.093, SRC = 3.522)
Do you notice food odors emanating from houses when you are outdoors?	Retained
When you are studying, or concentrated in general, do you get distracted by odors in the environment?	Retained
When you visit someone else’s house, do you notice how it smells?	Retained
Do you sniff at a new book?	Retained
When an acquaintance smells differently from normal, for example, because of a new perfume, do you immediately notice?	Removed due to overlap with item 8 (MI = 22.252,EPC = 0.155,SRC = 4.334)
Do you notice the smell of people’s breath or sweat?	Retained
Do you pay attention to the perfume, the aftershave or deodorant other people use?	Removed due to overlap with item 8 (MI = 27.256, EPC = 0.184, SRC = 4.641) and item 7 (MI = 18.712, EPC = 0.146, SRC = 3.96)
Are you the first one to smell gas?	Retained
Are you the first one to smell when the milk is sour?	Retained
Are you the first one to smell a fire, even when the smell only comes from a barbecue or fireplace?	Retained
Are you the first one to smell spoilt food in the fridge?	Removed due to overlap with item 11 (MI = 15.867, EPC = 0.096, SRC = 3.434)
Does an unpleasant smell in the environment that won’t go away make you anxious?	Retained
Do odors revive strong or vivid memories in you?	Removed due to standardized factor loading < 0.4
Do you sniff at clothes before you put them on?	Removed due to overlap with item 5 (MI = 19.695, EPC = 0.130, SRC = 3.678)
The smell of smoke or food is still lingering in your clothes from the night before. Do you put on new clothes because of the smell?	Retained
Does the smell of food sometimes put you off it?	Retained
When a room has an unpleasant smell, does it influence your mood?	Retained
When someone has an unpleasant body odor, does that make you find him or her unattractive? The body odor...	Retained
When someone has a pleasant body odor, do you find him or her attractive? The body odor...	Removed due to overlap with item 4 (MI = 13.352, EPC = 0.109, SRC = 3.058)
People differ in their sensitivity for odors. An unpleasant smell can leave one person unaffected yet be unbearable to another. How sensitive to odors do you think you are?	Removed due to standardized factor loading < 0.4
How important is it to you that your sheets smell fresh?	Retained
How important is it to you that your (future/potential) partner has a pleasant smell?	Removed due to overlap with item 10 (MI = 13.524, EPC = 0.090, EPC = 4.956)
Nowadays many cultivated flowers no longer have a fragrance. Do you find it important that flowers are fragrant?	Removed due to standardized factor loading < 0.4
How important are odors to you in your everyday life?	Removed due to overlap with item 19 (MI = 19.221, EPC = 0.141, SRC = 4.124)
You are in a public space sitting close to someone who has an unpleasant smell. Do you look for another seat if possible?	Removed due to overlap with item 23 (MI = 27.502, EPC = 0.166, SRC = 4.899)

Note: MI = Modification Indices, EPC = Expected Parameter Change, SRC = Standardized Residual Covariance.

#### 3.1.3. Step 3: Validating the shortened scale in a separate sample.

**Fig 2:**
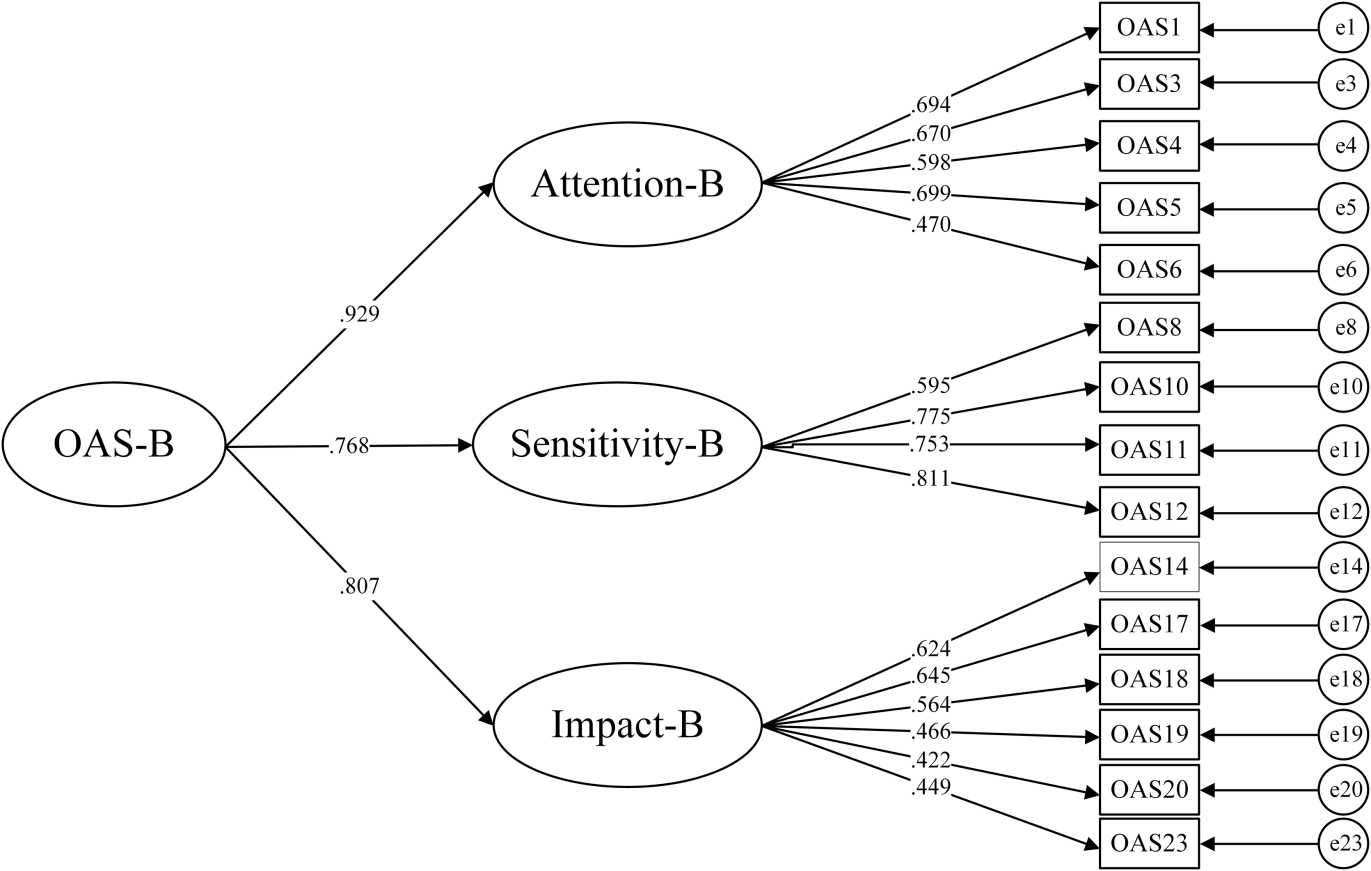
Confirmatory analysis structure of OAS-B with standardized factor loadings.

The reliability and validity of OAS-B were validated in Sample B. CFA showed the model fit indices for OAS-B as follows: $\chi^{\text{2}}/df$ = 2.594, RMSEA = 0.057, CFI = 0.938, TLI = 0.925, SRMR = 0.046, indicating good structural validity. Standardized factor loadings for all items in the scale were above 0.4 (see Fig 2). The Cronbach’s α coefficient for OAS-B was 0.868, with the Cronbach’s α coefficients for the subscales being: odor attention (0.765), odor sensitivity (0.799), and odor impact (0.763), demonstrating good internal consistency (see Table 2). Correlation analysis showed that the Pearson correlations between OAS-B and OAS, its subscales, SOS, and BOSQ ranged from moderate to high (*r* = 0.410 ~ 0.964, *p* < 0.01). Additionally, the correlations between OAS-B subscales and OAS, its subscales, and SOS were moderate to high (*r* = 0.411 ~ 0.978, *p* < 0.01), while correlations with BOSQ ranged from low to moderate (*r* = 0.285 ~ 0.373, *p* < 0.01), indicating good convergent validity for OAS-B. The correlations between OAS-B and GAD-7 were low (*r* = 0.122 ~ 0.165, *p* < 0.01), suggesting good discriminant validity (see Table 3). Items of OAS-B can been seen in the Supporting Information (S1 Appendix).

**Table 2:** Cronbach’s coefficients for OAS and OAS-B.

	Cronbach’s α coefficient		
	Sample A		Sample B
Dimensions	OAS	OAS-B	OAS-B
OAS	0.907	0.855	0.868
Attention	0.796	0.754	0.765
Sensitivity	0.867	0.816	0.799
Impact	0.827	0.723	0.763

Note: OAS = the Chinese version of the Odor Awareness Scale, OAS-B = the brief form of OAS.

**Table 3:** Correlations between OAS-B and other measures.

	1	2	3	4	5	6	7	8	9	10
OAS	--									
Attention	0.806**	--								
Sensitivity	0.865**	0.650**	--							
Impact	0.893**	0.567**	0.610**	--						
OAS-B	0.964**	0.841**	0.835**	0.825**	--					
Attention-B	0.804**	0.987**	0.657**	0.563**	0.845**	--				
Sensitivity-B	0.792**	0.615**	0.946**	0.525**	0.809**	0.622**	--			
Impact-B	0.781**	0.492**	0.508**	0.898**	0.810**	0.485**	0.440**	--		
SOS	0.600**	0.395**	0.524**	0.581**	0.518**	0.414**	0.457**	0.411**	--	
BOSQ	0.449**	0.349**	0.407**	0.395**	0.410**	0.363**	0.373**	0.285**	0.432**	--
GAD-7	0.161**	0.124**	0.126**	0.158**	0.165**	0.137**	0.122**	0.145**	0.095*	0.137**

Note: SOS = the Social Odor Awareness Scale, BOSQ = the Body Odor Sniffing Questionnaire, GAD-7 = the Generalized Anxiety Disorder Screener, OAS = the Chinese version of the Odor Awareness Scale, OAS-B = the brief form of OAS. *p < 0.05, **p < 0.01.

## 4. Discussion

This study employed a sequential item reduction strategy that integrates psychometric properties with the original theoretical model [21–23,35,36] to revise the Chinese version of the Odor Awareness Scale, resulting in a shortened version, OAS-B. Psychological tests are objective and standardized measures of behavioral samples [44], and the reliability and validity of a psychological test are crucial indicators of its scientific rigor [45]. OAS-B, by removing redundant items, enhances the reliability and validity of OAS, thereby increasing the scale’s equality. Compared to OAS, OAS-B improves model fit while preserving the conceptual model, retaining three factors: odor attention, odor sensitivity, and odor impact. In terms of internal consistency, OAS-B meets methodological standards. It also demonstrates good convergent and discriminant validity. Additionally, OAS-B reduced 12 items, representing a 44.4% reduction which falls within the range reported in a meta-analysis (21% − 88%) [21]. The reduced number of items in OAS-B facilitates practical application.

### 4.1. Item reduction

In this study, after removing items 15, 22, and 25 with standardized factor loadings below 0.4, we identified items with overlapping information based on indices and assessed their content validity to determine which items to remove. According to the information overlap indicators, items 2 (When someone is busy in the kitchen, do you notice the odor of the food being prepared?) and 3 (Do you notice food odors emanating from houses when you are outdoors?) have overlapping content regarding food and both belong to odor attention subscale. Since item 3 has a slightly higher standardized factor loading, we chose to retain it. Items 7 (When an acquaintance smells differently from normal, for example, because of a new perfume, do you immediately notice?), 9 (Do you pay attention to the perfume, the aftershave or deodorant other people use?), and 8 (Do you notice the smell of people’s breath or sweat?) have overlapping content regarding body odor and belong to odor sensitivity subscale, with item 8 having the highest factor loading, so we remained item 8. Items 11 (“Are you the first one to smell when the milk is sour?) and 13 (Are you the first one to smell spoilt food in the fridge?) have overlapping content related to food spoilage and belong to the odor sensitivity subscale. Since item 11 has a higher factor loading, it was retained. Items 21 (When someone has a pleasant body odor, do you find him or her attractive? The body odor…) and 4 (When you are studying, or concentrated in general, do you get distracted by odors in the environment?) have overlapping content but belong to different subscales. Since item 4 has a higher factor loading and odor attention subscale has fewer items, we retained item 4 in odor attention subscale. Items 24 (How important is it to you that your (future/potential) partner has a pleasant smell?) and 10 (Are you the first one to smell gas?) have overlapping content but belong to different subscales. Since item 10 has a better factor loading and odor sensitivity subscale has fewer items, we retained item 10 in odor sensitivity subscale. Items 26 (How important are odors to you in your everyday life?) and 19 (When a room has an unpleasant smell, does it influence your mood?) have overlapping content within the odor impact subscale with similar factor loadings. However, item 19 better fits the concept of the odor impact subscale, so we chose to retain it. Finally, items 27 (You are in a public space sitting close to someone who has an unpleasant smell. Do you look for another seat if possible?) and 23 (How important is it to you that your sheets smell fresh?) have overlapping content within odor impact subscale. Since item 23 has a higher factor loading and is shorter, which is crucial for maintaining participant engagement in lengthy surveys, we retained item 23.

Ultimately, we removed 12 items, retaining 15 items while preserving the three-factor structure consistent with OAS: odor attention subscale includes 5 items [1,3–6], odor sensitivity subscale includes 4 items [8,10–12], and odor impact subscale includes 6 items [14,17–20,23]. OAS-B demonstrated significantly improved model fit compared to the OAS, meeting methodological standards and achieving acceptable fit even in the validation subsample, indicating good structural validity. OAS-B retains the conceptual model structure of OAS while further enhancing its psychometric properties.

### 4.2. Reliability and validity

The Cronbach’s α coefficients for OAS and its subscales ranged from 0.796 to 0.907, consistent with previous research [20], indicating good internal consistency reliability. Although Cronbach’s α coefficient for OAS-B decreased compared to OAS, it is expected that Cronbach’s α typically decreases with fewer items [46–48]. Despite the decrease, the Cronbach’s α coefficients for OAS-B and its subscales all exceeded 0.7, demonstrating satisfactory internal consistency and reliability.

The convergent validity test revealed that the correlation coefficients between OAS-B and its subscales with OAS and its subscales range from 0.898 to 0.987, indicating a high degree of consistency in content between OAS-B and OAS. The correlations between SOS and OAS, as well as between SOS and OAS-B, are comparable and moderate, consistent with previous studies [31]. Conversely, the correlation coefficients between BOSQ and both OAS and OAS-B are similarly moderate and lower, in alignment with past research [20]. The discrepancy in correlation levels between SOS or BOSQ and OAS or OAS-B may be attributed to a bit of different constructs measured by SOS and BOSQ. Additionally, BOSQ uses a 4-point scale, whereas a 5-point scale is typically considered closer to a continuous variable [49], potentially accounting for the slightly lower Pearson correlation coefficients with OAS and OAS-B. Overall, OAS-B demonstrates significant correlations with other olfactory-related scales, generally ranging from moderate to high, highlighting its effective capture of olfactory information and affirming its strong convergent validity.

The Pearson correlation coefficients between GAD-7 and OAS-B, as well as its subscales, ranged from 0.122 to 0.165, comparable to the correlations between GAD-7 and OAS (*r* = 0.124 ~ 0.161), which is consistent with previous studies [20]. The low correlation indicates that GAD-7 and OAS-B measure different constructs. Therefore, OAS-B demonstrates good discriminant validity. Although the correlations between GAD-7 and OAS, OAS-B, SOS, and BOSQ are low, all correlations are statistically significant, aligning with prior research [9,20], which suggests a potential connection between anxiety symptoms and olfactory-related cognition. Extensive empirical work has converged on the conclusion that impaired olfactory performance covaries with mood-related psychopathologies such as major depressive and anxiety disorders [12–14,16]. Because the limbic circuitry that encodes chemosensory input simultaneously integrates emotional information from other modalities, it constitutes a neuroanatomical bridge through which olfactory experience can modulate—and be modulated by—emotional state [50]. Relative to controls, persons diagnosed with anxiety disorders consistently demonstrate diminished odor acuity [51], whereas individuals suffering from panic disorder report hypervigilance to ambient odor cues [17,18]. These divergent observations underscore the need for deeper investigation into how variations in odor awareness track the spectrum of affective dysregulation [52,53].

This study also has some limitations. The cross-sectional design with a convenience sampling method to recruit university students restricts the generalizability of the findings to broader, more diverse populations. Future research should assess the reliability and validity of the OAS-B across varied populations, particularly in terms of clinical or environmental contexts. Besides, the item reduction in the OAS-B may result in some loss of information compared to the OAS. Researchers should carefully select the version that best aligns with their specific research objectives. There may be a potential limitation in that using self-reported measures to assess odor awareness may cause recall bias. Future studies could investigate the OAS-B’s test-retest reliability and its sensitivity to change, explore the scale’s invariance across more demographic factors, and examine the relationship between OAS-B scores and other relevant variables (e.g., olfactory performance, health outcomes).

In conclusion, we successfully revised OAS into a brief version using the sequential item reduction strategy. The revision involved rigorous validity and reliability testing, which demonstrated that OAS-B significantly improves upon the psychometric properties of OAS. OAS-B exhibits strong internal consistency, structural validity, convergent validity, and discriminant validity. Its moderate length and concise content make it an effective tool for measuring odor awareness in general populations.

## Supporting information

S1 Appendix The brief form of the Chinese version of the Odor Awareness Scale. (DOCX)

## References

[1] Baron RA. Environmentally Induced Positive Affect: Its Impact on Self‐Efficacy, Task Performance, Negotiation, and Conflict1. J Applied Social Pyschol. 1990;20(5):368–84. doi: 10.1111/j.1559-1816.1990.tb00417.x

[2] Wrzesniewski A, McCauley C, Rozin P. Odor and affect: individual differences in the impact of odor on liking for places, things and people. Chem Senses. 1999;24(6):713–21. doi: 10.1093/chemse/24.6.713 10587506

[3] Fiore AM, Yah X, Yoh E. Effects of a product display and environmental fragrancing on approach responses and pleasurable experiences. Psychol Mark. 2000;17(1):27–54. doi: 10.1002/(sici)1520-6793(200001)17:1<27::aid-mar3>3.0.co;2-c

[4] Larsson M, Willander J. Autobiographical odor memory. Ann N Y Acad Sci. 2009;1170:318–23. doi: 10.1111/j.1749-6632.2009.03934.x 19686154

[5] Ehrlichman H, Bastone L. The use of odour in the study of emotion. In: Fragrance: The psychology and biology of perfume. New York, NY, US: Elsevier Applied Science Publishers/Elsevier Science Publishers; 1992. p. 143–59.

[6] Smeets MAM, Schifferstein HNJ, Boelema SR, Lensvelt-Mulders G. The Odor Awareness Scale: a new scale for measuring positive and negative odor awareness. Chem Senses. 2008;33(8):725–34. doi: 10.1093/chemse/bjn038 18622009

[7] Dal Bò E, Natali L, Gentili C, Cecchetto C. Low odor awareness predicts reduced olfactory abilities in women with depressive symptoms, but not with anxiety symptoms. J Affect Disord. 2023;338:171–9. doi: 10.1016/j.jad.2023.06.009 37290528

[8] Cecchetto C, Dal Bò E, Aiello M, Fischmeister FPS, Gentili C, Osimo SA. Alexithymia modulates the attitudes towards odors but not the olfactory abilities or the affective reactions to odors. PLoS One. 2023;18(6):e0278496. doi: 10.1371/journal.pone.0278496 37279254 PMC10243640

[9] Dal Bò E, Gentili C, Castellani A, Tripodi C, Fischmeister FPS, Cecchetto C. Olfactory meta-cognition in individuals with depressive and anxiety symptoms: The differential role of common and social odors. J Affect Disord. 2022;308:259–67. doi: 10.1016/j.jad.2022.04.071 35429542

[10] Nováková L, Varella Valentova J, Havlíček J. Engagement in Olfaction-Related Activities is Associated with the Ability of Odor Identification and Odor Awareness. Chem Percept. 2014;7(2):56–67. doi: 10.1007/s12078-014-9167-2

[11] Arshamian A, Willander J, Larsson M. Olfactory awareness is positively associated to odour memory. J Cognit Psychol. 2011;23(2):220–6. doi: 10.1080/20445911.2011.483226

[12] Kamath V, Paksarian D, Cui L, Moberg PJ, Turetsky BI, Merikangas KR. Olfactory processing in bipolar disorder, major depression, and anxiety. Bipolar Disord. 2018;20(6):547–55. doi: 10.1111/bdi.12625 29441710

[13] Kohli P, Soler ZM, Nguyen SA, Muus JS, Schlosser RJ. The Association Between Olfaction and Depression: A Systematic Review. Chem Senses. 2016;41(6):479–86. doi: 10.1093/chemse/bjw061 27170667 PMC4918728

[14] Mattos JL, Schlosser RJ, Storck KA, Soler ZM. Understanding the relationship between olfactory-specific quality of life, objective olfactory loss, and patient factors in chronic rhinosinusitis. Int Forum Allergy Rhinol. 2017;7(7):734–40. doi: 10.1002/alr.21940 28519966 PMC5751751

[15] Moberg PJ, Agrin R, Gur RE, Gur RC, Turetsky BI, Doty RL. Olfactory dysfunction in schizophrenia: a qualitative and quantitative review. Neuropsychopharmacology. 1999;21(3):325–40. doi: 10.1016/S0893-133X(99)00019-6 10457530

[16] Taalman H, Wallace C, Milev R. Olfactory Functioning and Depression: A Systematic Review. Front Psychiatry. 2017;8:190. doi: 10.3389/fpsyt.2017.00190 29033860 PMC5627007

[17] Burón E, Bulbena A, Bulbena-Cabré A. Olfactory functioning in panic disorder. J Affect Disord. 2015;175:292–8. doi: 10.1016/j.jad.2015.01.049 25661394

[18] Burón E, Bulbena A, Bulbena-Cabré A, Rosado S, Pailhez G. Both anxiety and joint laxity determine the olfactory features in panic disorder. Psychiatry Res. 2018;262:420–6. doi: 10.1016/j.psychres.2017.09.018 28923431

[19] Zhang B, Li X, Deng H, Tan P, He W, Huang S, et al. The relationship of personality, alexithymia, anxiety symptoms, and odor awareness: a mediation analysis. BMC Psychiatry. 2024;24(1):185. doi: 10.1186/s12888-024-05653-y 38448836 PMC10916267

[20] Zhang B, Li X, Tan P, Liu Y, He W, Wang L, et al. Factor structure and psychometric properties of the Chinese version of the Odor Awareness Scale. Front Psychiatry. 2023;14:1228179. doi: 10.3389/fpsyt.2023.1228179 37575563 PMC10415028

[21] Goetz C, Coste J, Lemetayer F, Rat A-C, Montel S, Recchia S, et al. Item reduction based on rigorous methodological guidelines is necessary to maintain validity when shortening composite measurement scales. J Clin Epidemiol. 2013;66(7):710–8. doi: 10.1016/j.jclinepi.2012.12.015 23566375

[22] Stanton JM, Sinar EF, Balzer WK, Smith PC. Issues and strategies for reducing the length of self‐report scales. Personnel Psychol. 2002;55(1):167–94. doi: 10.1111/j.1744-6570.2002.tb00108.x

[23] Smith GT, McCarthy DM, Anderson KG. On the sins of short-form development. Psychol Assess. 2000;12(1):102–11. doi: 10.1037//1040-3590.12.1.102 10752369

[24] Wang M. Latent variable modeling and mplus application: basic part. Chongqing: Chongqing University Press; 2014.

[25] Geiser C, editor. Data analysis with Mplus. Guilford Press; 2012.

[26] Kelloway EK, editor. Using Mplus for structural equation modeling: A researcher’s guide. Sage Publications; 2014.

[27] Wang J, Wang X, editors. Structural equation modeling: Applications using Mplus. John Wiley & Sons; 2019.

[28] Goertz G. Social science concepts: A user’s guide. Princeton University Press; 2006.

[29] Oleszkiewicz A, Schriever VA, Croy I, Hähner A, Hummel T. Updated Sniffin’ Sticks normative data based on an extended sample of 9139 subjects. Eur Arch Otorhinolaryngol. 2019;276(3):719–28. doi: 10.1007/s00405-018-5248-1 30554358 PMC6411676

[30] MacCallum RC, Widaman KF, Preacher KJ, Hong S. Sample Size in Factor Analysis: The Role of Model Error. Multivariate Behav Res. 2001;36(4):611–37. doi: 10.1207/S15327906MBR3604_06 26822184

[31] Dal Bò E, Gentili C, Spoto A, Bruno G, Castellani A, Tripodi C, et al. The social odor scale: Development and initial validation of a new scale for the assessment of social odor awareness. PLoS One. 2021;16(12):e0260587. doi: 10.1371/journal.pone.0260587 34905551 PMC8670672

[32] Li Z-L, Yue Qi, Mahmut MK, Zou L-Q. Do you often sniff yourself or others? Development of the Body Odor Sniffing Questionnaire and a cross-cultural survey in China and the USA. Physiol Behav. 2022;255:113934. doi: 10.1016/j.physbeh.2022.113934 35908610

[33] Spitzer RL, Kroenke K, Williams JBW, Löwe B. A brief measure for assessing generalized anxiety disorder: the GAD-7. Arch Intern Med. 2006;166(10):1092–7. doi: 10.1001/archinte.166.10.1092 16717171

[34] Xiaoyan H, Chunbo L, Jie Q, Haisong C, Wenyuan W. Reliability and validity of a generalized anxiety disorder scale in general hospital outpatients. Shanghai Arch Psych. 2010;22(4):200–3.

[35] Sexton E, King-Kallimanis BL, Morgan K, McGee H. Development of the brief ageing perceptions questionnaire (B-APQ): a confirmatory factor analysis approach to item reduction. BMC Geriatr. 2014;14:44. doi: 10.1186/1471-2318-14-44 24716631 PMC4021231

[36] Shahhosseini Z, Nikbakht R, Motaghi Z, Hosseini Tabaghdehi M. Development of the short form Iranian women childbirth experience questionnaire: a confirmatory factor analysis approach item reduction. BMC Pregnancy Childbirth. 2023;23(1):48. doi: 10.1186/s12884-023-05378-y 36670388 PMC9854137

[37] Slotman A, Cramm JM, Nieboer AP. Validation of the Dutch Aging Perceptions Questionnaire and development of a short version. Health Qual Life Outcomes. 2015;13:54. doi: 10.1186/s12955-015-0248-y 25963849 PMC4426604

[38] Muthén B, Muthén L. Mplus. In: Handbook of item response theory. Chapman and Hall/CRC; 2017. p. 507–18.

[39] Ford JK, MacCallum RC, Tait M. The application of exploratory factor analysis in applied psychology: a critical review and analysis. Personnel Psychol. 1986;39(2):291–314. doi: 10.1111/j.1744-6570.1986.tb00583.x

[40] Schermelleh-Engel K, Moosbrugger H, Müller H. Evaluating the fit of structural equation models: Tests of significance and descriptive goodness-of-fit measures. Method Psychol Res Online. 2003;8(2):23–74.

[41] Hu L, Bentler PM. Cutoff criteria for fit indexes in covariance structure analysis: Conventional criteria versus new alternatives. Struct Equat Model Multidiscip J. 1999;6(1):1–55. doi: 10.1080/10705519909540118

[42] Schumacker RE, Lomax RG. A beginner’s guide to structural equation modeling. Psychology Press; 2004.

[43] Byrne BM. Structural equation modeling with EQS: Basic concepts, applications, and programming. 2nd ed. London: Routledge; 2013.

[44] Anastasi A, Urbina S, editors. Psychological testing. 7th ed. Prentice Hall/Pearson Education; 1997.

[45] Li G. Psychological measurement. Beijing: Tsinghua University Press; 2019.

[46] Tavakol M, Dennick R. Making sense of Cronbach’s alpha. Int J Med Educ. 2011;2:53–5. doi: 10.5116/ijme.4dfb.8dfd 28029643 PMC4205511

[47] Nunnally JC, Bernstein IH. Psychometric theory. 3rd ed. New York: McGraw-Hill Higher; 1994.

[48] Streiner DL. Starting at the beginning: an introduction to coefficient alpha and internal consistency. J Pers Assess. 2003;80(1):99–103. doi: 10.1207/S15327752JPA8001_18 12584072

[49] Dolan CV. Factor analysis of variables with 2, 3, 5 and 7 response categories: A comparison of categorical variable estimators using simulated data. Brit J Math Statis. 1994;47(2):309–26. doi: 10.1111/j.2044-8317.1994.tb01039.x

[50] Soudry Y, Lemogne C, Malinvaud D, Consoli S-M, Bonfils P. Olfactory system and emotion: common substrates. Eur Ann Otorhinolaryngol Head Neck Dis. 2011;128(1):18–23. doi: 10.1016/j.anorl.2010.09.007 21227767

[51] Clepce M, Reich K, Gossler A, Kornhuber J, Thuerauf N. Olfactory abnormalities in anxiety disorders. Neurosci Lett. 2012;511(1):43–6. doi: 10.1016/j.neulet.2012.01.034 22306090

[52] Croy I, Nordin S, Hummel T. Olfactory disorders and quality of life--an updated review. Chem Senses. 2014;39(3):185–94. doi: 10.1093/chemse/bjt072 24429163

[53] Sanna F, Loy F, Piras R, Moat A, Masala C. Age-Related Cognitive Decline and the Olfactory Identification Deficit Are Associated to Increased Level of Depression. Front Neurosci. 2021;15:599593. doi: 10.3389/fnins.2021.599593 33692667 PMC7937898

