# Supplementary material for: Development of a brief form of the Chinese version of the Odor Awareness Scale(OAS-B): A confirmatory factor analysis approach to item reduction
Source: PLoS One. 2025 Oct 9;20(10):e0334168. doi: 10.1371/journal.pone.0334168 (PMC12510569; doi:10.1371/journal.pone.0334168)
Supplement: S1 Appendix — (DOCX) [file pone.0334168.s001.docx]

**Supporting information**

# The brief form of the Chinese version of the Odor Awareness Scale

| 1. | When you walk through the woods, do you pay attention to the odors surrounding you? | | 1.never | 2.seldom | 3.sometimes | 4.often | 5.always |
| --- | --- | --- | --- | --- | --- | --- | --- |
| 2. | Do you notice food odors emanating from houses when you are outdoors? | | 1.never | 2.seldom | 3.sometimes | 4.often | 5.always |
| 3. | When you are studying, or concentrated in general, do you get distracted by odors in the environment? | | 1.never | 2.seldom | 3.sometimes | 4.often | 5.always |
| 4. | When you visit someone else’s house, do you notice how it smells? | | 1.never | 2.seldom | 3.sometimes | 4.often | 5.always |
| 5. | Do you sniff at a new book? | | 1.never | 2.seldom | 3.sometimes | 4.often | 5.always |
| 6. | Do you notice the smell of people’s breath or sweat? | | 1.never | 2.seldom | 3.sometimes | 4.often | 5.always |
| 7. | Are you the first one to smell gas? | | 1.never | 2.seldom | 3.sometimes | 4.often | 5.always |
| 8. | Are you the first one to smell when the milk is sour? | | 1.never | 2.seldom | 3.sometimes | 4.often | 5.always |
| 9. | Are you the first one to smell a fire, even when the smell only comes from a barbecue or fireplace? | | 1.never | 2.seldom | 3.sometimes | 4.often | 5.always |
| 10. | Does an unpleasant smell in the environment that won’t go away make you anxious? | | 1.never | 2.seldom | 3.sometimes | 4.often | 5.always |
| 11. | The smell of smoke or food is still lingering in your clothes from the night before. Do you put on new clothes because of the smell? | | 1.never | 2.seldom | 3.sometimes | 4.often | 5.always |
| 12. | Does the smell of food sometimes put you off it? | | 1.never | 2.seldom | 3.sometimes | 4.often | 5.always |
| 13. | When a room has an unpleasant smell, does it influence your mood?__________ | 1. (almost) no influence 2. a little influence 3. some influence 4. much influence 5. Has very much influence | | | | | |
| 14. | When someone has an unpleasant body odor, does that make you find him or her unattractive? The body odor _______ | 1. (almost) no influence 2. a little influence 3. some influence 4. much influence 5. Has very much influence | | | | | |
| 15. | How important is it to you that your sheets smell fresh? | 1. not important at all | 2. slightly important | 3. moderately important | 4.important | 5. very important | |
